# A Practice Much Affected

**Published:** 1896-09

**Authors:** 


A PRACTICE much affected by certain persons is to "pooh pooh" arguments they cannot answer, and denounce as trash facts that cannot be controverted. Our Houston neighbor with the *away out yonder name*, says: "This is a progressive age, and the progressive physician no longer takes any notice of such 'wailing and nashing [*sic*] of teeth,' as is indulged in by the 'Red Back' to the extent of eighteen pages, against the action of the State Medical Society. Let us throw prejudice, malice and old animosities aside and be men, thinking men, and raise medicine to a higher plane."

That is *precisely what we advocate;* hence we *protest* against *degrading* medicine to the level of a "pathy" or a "school of medicine," and making common cause with homeopaths by recognizing them. The *S. W. Medical Record* has queer ideas of progress if the step contemplated by the State Medical Association (recognizing homeopaths) be taken as an illustration of it. It is progressing *backward* after the manner of a crab. As to "wailing and [g]nashing of teeth" why should *we* "wail" or "gnash?" It's not *our* funeral; we do not aspire to the dignity, even, of being a chief mourner. Readers are ieferred to Dr. Maclean's letter in this issue. Dr. Red and his colleagues will find there a fine quality of what they call "wail" and [g]"nash."

We would remark, parenthetically, that, Dr. Red, to the contrary notwithstanding, the "progressive physicians" *appear* to take a *great deal* of notice of what the "Red Back" says, *on every subject.* Even as "progressive" a physician as Dr. Red (or the writer of the paragraph quoted) "noticed" the decried eighteen pages of wailing and "nashing" (gnashing) and the *Texas Medical News* devoted an almost entire edition to it.

